# Fish Skin Grafts Versus Alternative Wound Dressings in Wound Care: A Systematic Review of the Literature

**DOI:** 10.7759/cureus.36348

**Published:** 2023-03-19

**Authors:** Mohamed Ibrahim, Haneen S Ayyoubi, Layth A Alkhairi, Hozaifa Tabbaa, Isaac Elkins, Ravish Narvel

**Affiliations:** 1 College of Osteopathic Medicine, Lake Erie College of Osteopathic Medicine, Bradenton, USA; 2 Internal Medicine, Barry University, St. Petersburg, USA; 3 Internal Medicine, Ascension St. Vincent's Riverside Hospital, Jacksonville, USA

**Keywords:** burn wounds, foot ulcer, dehydrated human amnion/chorion membrane allograft, xenografts, acellular fish skin, fish skin graft

## Abstract

Wound healing poses a variety of challenges making it a vital subject in medicine. With the advancement of science, we have seen the use of a new xenograft known as acellular fish skin (AFS) grafts that are derived from either Atlantic cod or Nile Tilapia. Fish skin has shown anti-inflammatory and anti-bacterial properties that support and improve wound healing in a variety of wounds including burns and diabetic foot ulcers (DFU). There is ongoing research that evaluates the efficacy of fish skin grafts in comparison to alternative wound healing techniques. A literature search was conducted through the National Library of Medicine with search terms fish skin graft, AFS, xenograft, dehydrated human amnion/chorion, ulcer, burns, and wounds. A total of ten studies that investigate the efficacy of fish skin grafts either in comparison to a different wound healing technique or by simply observing wound healing with fish skin grafts and recording the results were chosen. AFS showed superior healing in comparison to collagen alginate dressings, silver sulfadiazine cream 1%, and allografts. Although there is no one specific gold standard technique for wound healing, fish skin grafts demonstrated overall improved and quicker wound healing, fewer dressing changes, less pain, and lower costs.

## Introduction and background

Burns and chronic foot ulcers are just two types of wounds that demonstrate a challenge for both healthcare providers and patients. Chronic foot ulcers are often seen in diabetics and unfortunately, are a common cause of amputations [1]. Burns are a leading cause of morbidity and occur more commonly in middle- and low-income countries [1]. In general, wound healing may be complicated by graft rejections, graft-transmitted diseases, infections, pain, and substantial socioeconomic and treatment-related costs. Acellular fish skin (AFS) grafts have emerged as a potentially cost-effective wound treatment method with improved wound healing outcomes [2]. The purpose of this review is to compare the efficacy of AFS grafts with other alternative wound treatment methods.

As the largest organ in our body, our skin plays a vital role as a first-line defense for our health and well-being. However, when that barrier is penetrated and the healing process is interrupted, it is critical to restore that protection before chronic complications occur and become detrimental to one’s health. Wounds are often the result of accidents, traumas, or pathological causes and in certain situations may become chronic and difficult to heal, as in the case of diabetic foot ulcers (DFU). Other times, wounds can encompass considerable segments of the body making treatment difficult when insufficient amounts of donor skin are available [1,2]. Decreased quality of life and large healthcare costs are major consequences of burns and chronic wounds [3,4].

There are numerous ways to treat wounds depending on their depth and extent of damage including, but not limited to, collagen alginate dressings, silver sulfadiazine cream 1%, autografts, allografts, and xenografts. Collagen alginate dressing is a collagen-rich, sterile dressing that provides a moist environment for wounds in order to support granulation and epithelization of tissue thus permitting more effective wound healing [1]. Silver sulfadiazine cream 1% is used to create a barrier between a wound and the environment as well as provide anti-bacterial properties which allow for re-epithelization and healing [2]. Autografts are skin grafts that are transferred from the same person with the wound but from a different, healthy location on the body. Allografts are skin grafts that are transferred from a different person also known as a donor and xenografts are transferred from an animal such as pigs, cattle, or fish [4]. These grafts are termed as cellular and/or tissue-based products (CTP) [1,4].

It is notable to discuss some of the limitations of these grafts. For instance, the use of collagen alginate dressings or silver sulfadiazine cream requires frequent dressing changes which can be difficult for both healthcare providers and the patients [2]. Autografts require the formation of a new wound on a different part of the body resulting in a concern for surgical complications or morbidity and may not even be an option in large wounds or burns. Allografts carry a risk for graft rejection due to the unfortunate potential for the host’s immune system to identify the donor skin as foreign and proceed to attack it; although, most modern-day human allograft material is made from an acellular dermal matrix that is designed not be recognized by the host’s immune system [1]. Xenografts derived from mammalian sources carry the risk of transmitting viral diseases requiring these grafts to undergo expensive processing with powerful detergents to decrease that risk [1].

The structure of AFS grafts is unique and has proved to be advantageous in regenerative medicine. Firstly, it shows some similarities in structure to human skin, making it a compatible alternative with the ability to promote cellular proliferation in wound healing without hypersensitivity reactions as proven in the included studies that show a 0% reaction rate amongst the patient participants being treated for chronic wounds [1,4]. The most exceptional property of AFS grafts that make it efficacious is its lipid profile. Its lipid profile is rich in omega-3 polyunsaturated fatty acids with a large concentration of docosahexaenoic acid (DHA) and eicosapentaenoic acid (EPA), which are associated with anti-microbial and anti-inflammatory properties even against Methicillin-resistant Staphylococcus aureus (MRSA). This allows for a more regulated inflammatory response and possible faster wound healing. Another beneficial quality of AFS grafts is the fact that it does not carry the risk of viral disease transmission therefore it does not require harsh processing with powerful detergents allowing it to retain more of its unique structure and properties as well as making it more cost effective [1,4]. AFS grafts are also very porous, having about 16.7 large diameter apertures for every 100 µm2 allowing it to properly adhere to human skin and promote the passage of human fibroblasts, which are known to play an important role in effective wound healing. These unique properties of AFS grafts make this xenograft an advantageous option in wound management and healing and is worth exploring [1,4].

## Review

Methods

The articles included in Table 1 below are peer-reviewed studies researched via an online medical journal database known as PubMed. Keywords such as “fish skin graft, acellular fish skin, and wound grafts” were used to populate a total of 241 results. To ensure relativity and applicability to modern medicine, only articles that were published in the last five years were chosen. This initial screening excluded 161 articles with 80 meeting our set requirements. For the 80 selected articles, the inclusion criteria consisted of randomized control trials (RCTs), retrospective studies, and preclinical trials that compared AFS grafts with CTPs or other standard controls. The full availability and accessibility of the articles were also used as a screening method to exclude certain options. The references of the chosen papers were surveyed and used to find other pertinent studies that more consistently assessed AFS grafts against other wound/ulcer treatment options. The included articles were organized thematically based on the type of alternative wound care used in comparison to AFS grafts. Within the themes, the studies were organized chronologically. The literature was categorized as follows: an observational retrospective study that observed wound healing with the use of AFS grafts, a randomized controlled double-blind study, a preclinical trial and case-control retrospective study that compared AFS grafts with other CTPs, three randomized controlled trials that compared AFS grafts with silver sulfadiazine cream 1% in burn wounds, and a retrospective comparative cohort study in addition to two randomized controlled trials that compared AFS grafts with collagen alginate dressings in DFUs.

**Table 1:** Summary of Included Studies and Result Analysis AFS: acellular fish skin; dHACM: dehydrated human amnio‐chorionic membrane; DPT: deep partial thickness; STSG; split thickness skin graft; FBD: fetal bovine dermis; SPT: superficial partial thickness; SSDC: silver sulfadiazine cream 1%; DFU: diabetic foot ulcer; SOC: standard of care

Studies	Year	Study type	Test performed	Results
Michael S, Winters C, Khan M. [5]	2019	Retrospective study	AFS grafts, percentage of wound surface closure	Over half the wounds healed completely
Kirsner RS, Margolis DJ, Baldursson BT, et al. [6]	2019	Randomized controlled double-blind study	Full thickness wounds created on volunteers and treated with either AFS grafts or dHACM	Wounds treated with AFS grafts healed faster.
Stone II R, Saathoff EC, Larson DA, et al. [7]	2021	Preclinical trial	DPT wounds created on pigs and treated with either AFS graft or FBD	Wounds treated with AFS grafts healed faster compared to FBD grafts
Wallner C, Holtermann J, Drysch M, et al. [8]	2022	Retrospective case control study	SPT burns treated with Suprathel, DPT burns treated with AFS graft or a STSG	Wounds treated with AFS grafts healed faster than Suprathel or STSG
Lima Júnior EM, Moraes Filho MO, Forte AJ, et al. [9]	2019	Randomized controlled trial	SPT burn wounds treated with AFS grafts or SSDC	Burn wounds healed at the same rate with AFS graft and silver sulfadiazine cream.
Lima Júnior EM, De Moraes Filho MO, Costa BA, et al. [10]	2020	Randomized controlled trial	Superficial and DPT burn wounds treated with either AFS grafts or SSDC	Burn wounds treated with AFS grafts healed faster, required fewer dressing changes and less analgesic requirements
Lima Júnior EM, de Moraes Filho MO, Costa BA, et al. [11]	2021	Randomized controlled trial	SPT burn wounds treated with AFS grafts or SSDC in an outpatient setting	Burn wounds treated with AFS grafts healed faster, were less painful and had lower treatment costs
Winters C, Kirsner RS, Margolis DJ, Lantis JC. [12]	2020	Retrospective comparative cohort study	Cost effectiveness of DFU treatment with AFS grafts compared to SOC treatment	Reduced costs when wounds are treated with AFS grafts
Lullove EJ, Liden B, Winters C, McEneaney P, Raphael A, Lantis Ii JC. [13]	2021	Randomized controlled trial	Treatment resistant DFUs treated with collagen alginate dressing with or without AFS graft	Significantly higher number of complete wound healing seen in DFUs treated with AFS grafts
Lullove EJ, Liden B, McEneaney P, et al. [14]	2022	Randomized controlled trial	DFUs treated with either collagen alginate dressing with or without AFS graft	More wound closures seen in with AFG treatment.

**Figure 1:**
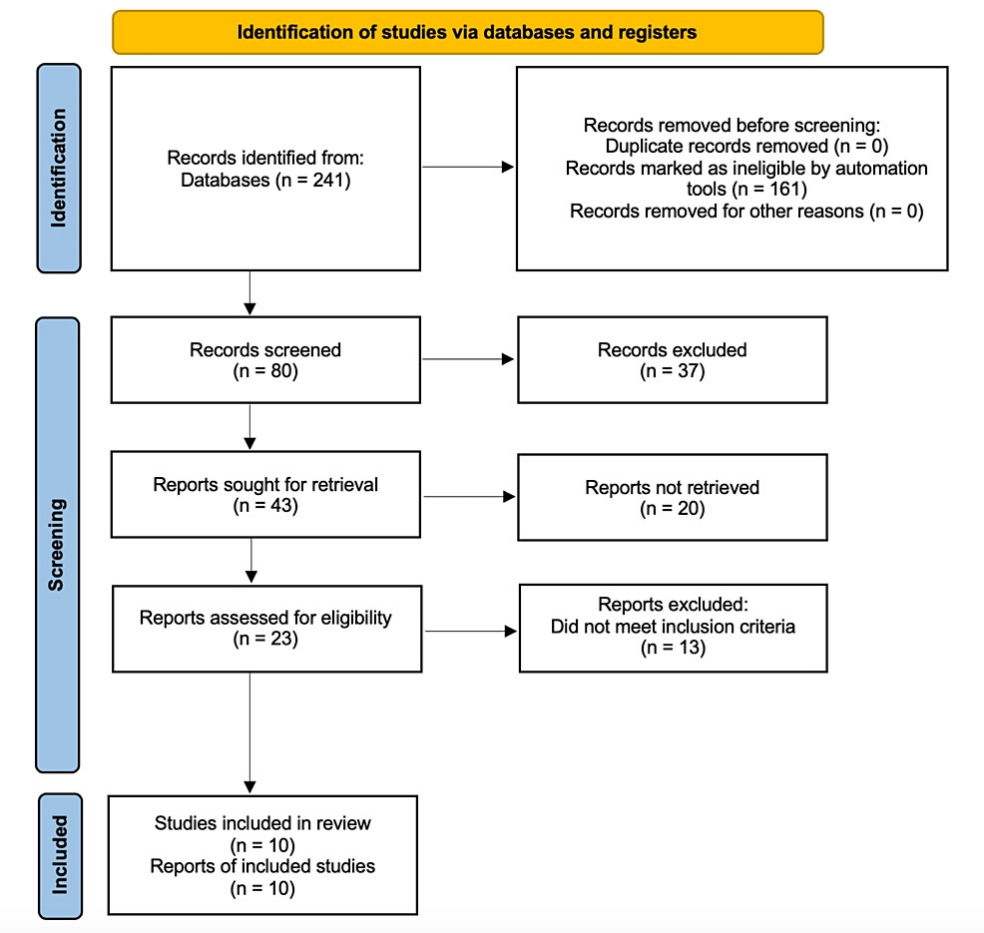
PRISMA Flow Diagram PRISMA: Preferred Reporting Items for Systematic Reviews and Meta-Analyses

Preferred Reporting Items for Systematic Reviews and Meta-Analyses (PRISMA) guidelines were used to conduct this systematic review of literature in order to enhance the goal and objective of this study. The associated PRISMA flowchart is shown below in Figure 1. 

Results

Retrospective Observational Study Evaluating Percentage of Wound Closure

In 2019, Michael et al. performed a retrospective study evaluating the percentage of wound closures in DFU after 16 weeks of treatment with AFS grafts [5]. They selected 51 patients and had a total of 58 DFUs. The wounds were first debrided then a portion of the ASF graft was placed, and the wound was dressed. At the 16th week mark, 35 of the 58 wounds had completely healed (60.34%) with an average decrease in wound surface area of 87.57% [5]. After 16 weeks, 43 DFUs had >90% decrease in wound surface area and 49 DFUs had >75% decrease in wound surface area. Of the 58 wounds, two wounds did not show any decrease in wound surface area at the 16th-week mark; however, one of the DFUs required two extra applications of the AFS graft and healed at 24 weeks while the other DFU required three more episodes of wound debridement until it healed but did not require additional applications of the AFS grafts [5]. It is notable to mention that the last wound was the oldest wound being present for over three years. On average, the wounds required 4.9 AFS graft applications to heal and the first fully healed 35 wounds mentioned earlier, averaged a mere ten weeks to achieve complete wound healing [5]. The study concluded that AFS grafts permit for faster wound healing and allowed DFUs, with the addition of debridement, to properly transition from a chronic treatment-resistant state to an acute pro-inflammatory state with the support of AFS grafts as suggested by previous research [5].

Studies Comparing AFS Grafts With Cellular And/Or Tissue-Based Products 

Kirsner et al. conducted a randomized controlled double-blind study in 2019 comparing the use of AFS grafts and dehydrated human amnio‐chorionic membrane (dHACM) by creating two full thickness punch biopsy wounds four mm in diameter on healthy volunteers. The acute wounds were meant to mimic freshly debrided chronic wounds prior to treatment [6]. On every individual one wound was treated with an AFS graft while the other was treated with a dHACM allograft [6]. There were 85 patients with a total of 170 wounds and the patient along with the physician evaluating the wounds were blinded to which wound was treated with which CTP. The patients were followed up for a total of seven times over a 28-day period and the wounds were evaluated for any signs of infection, wide areas of erythema, pain level and any new signs of bleeding as well as whether the wound had healed or not [6]. On day 14 it was noted that the wounds treated with AFS were healing more rapidly than the wounds treated with dHACM. Twenty-two days were required for the wounds treated with AFS graft to heal while the dHACM arm only required about 24 days to heal. No infections were noted throughout the study; however, four patients developed surrounding erythema greater than one mm in both their wounds while two patients had erythema of the same classification in the wound treated with the AFS graft only [6]. Since the difference was minor it was not recognized as being caused by either treatment type. The study concluded that the wounds treated with AFS grafts healed at a faster rate in comparison to the wounds treated with dHACM but showed no difference in adverse reactions in both arms. They also considered the difference in cost of treatment per arm noting that wounds treated with dHACM were 76% more costly than wounds treated with an AFS graft [6].

A preclinical trial comparing the efficacy of AFS grafts with fetal bovine dermis (FBD) grafts on deep partial thickness burn wounds was performed by Stone II R et al in 2021. performed. Twenty-four deep partial thickness burn wounds 5 x 5 mm in diameter were surgically created on six anesthetized Yorkshire pigs [7]. The next day the wounds were debrided and treated with either AFS grafts or FBD grafts and the AFS grafts were reapplied after seven days. The wounds were evaluated a total of six times over a 62-day period for size of surface area, level of hydration, amount of re-epithelization and trans-epidermal water loss [7]. On the seventh day, it was noted that the AFS grafts had integrated into the wound at a quicker rate in comparison to the FBD graft and after the second application of the AFS on day seven. Wounds treated with AFS grafts showed quicker re-epithelization and contraction rates in comparison to FBD grafts, which assessed the level of wound closure. The surface area of the wound was significantly reduced in wounds treated with AFS grafts in comparison to the FBD grafts on day 14 [7]. By day 28, the wounds treated with either arm began to contract at a similar rate. The best way to assess the skin’s return to normal function was by measuring the level of hydration and trans-epidermal water loss in each group [7]. On day 21, it was noted that the level of hydration was significantly lower in wounds treated with AFS grafts (309.7 µS) in comparison to FBD grafts (2500.4 µS); however, the trans-epidermal water loss showed no difference between the two groups. Normal skin function returned almost completely by day 60 in both groups [7]. Laser speckle contrast imaging (LSI) was used to illustrate the level of blood flow of each wound. On day 14, wounds treated with AFS grafts showed significantly increased blood flow with LSI in comparison to wounds treated with FBD grafts. Overall, this study demonstrates the superior wound healing properties of AFS grafts over FBD grafts for the treatment of deep partial thickness wounds [7].

Wallner et al. performed a retrospective case-control study to determine the best CPT for treatment of mixed dermal burn wounds in 2022. Twelve patients were selected to be followed for 12 months after the burn injury [8]. Their wounds were first debrided enzymatically one day after injury then superficial partial-thickness burns were treated with Suprathel, a synthetic skin substitute that acts like a CTP, while deep partial thickness burns were treated with either an autologous STSG or an AFS graft [8]. A total of 12 wounds were treated with AFS grafts, seven wounds treated with autologous STSG, and eight wounds treated with Suprathel. Epithelialization occurred on day 22 (± 6.3) in wounds treated with AFS grafts, on day 34.7 (± 12.5) in wounds treated with autologous STSG, and on day 45.6 (± 6.6) in wounds treated with Suprathel. Elasticity was also assessed 12 months after burn injury by using a Cutometer [8]. The values of relative maximal deformation are as follows: AFS graft (102.3% ± 14.3%), autologous STSG (66% ± 9.1%), and Suprathel (41.9% ± 4.8%) in comparison to what is normally found in healthy skin [8]. This translates to significantly improved regeneration and elasticity 12 months after injury in patients treated with AFS grafts. A sebumeter and corneometer were used to assess the regenerated skin’s water and sebum content. For sebum content, ASF grafts ranked higher 119.3% (± 17.6%) in comparison to autologous STSG 74.1% (± 16.3%) and Suprathel 45.4% (± 15.2%). For water content, AFS grafts also ranked higher 96.9% (± 7.1%) in comparison to autologous STSG 64.5% (± 6.4%) and Suprathel 53.1% (± 4.4%). This concludes that the use of AFS grafts in burn wounds proves to show faster wound healing, improved long-term functionality, and even enhanced aesthetic results in comparison to synthetic skin substitutes and autologous STSG [8].

Studies Comparing AFS Grafts With Silver Sulfadiazine Cream 1%

In 2019, Lima Junior et al. conducted a randomized control trial to evaluate the efficacy of AFS grafts in comparison to silver sulfadiazine cream 1% in the treatment of superficial partial thickness burns [9]. The study consisted of 30 children between the ages of two and 12 years old who were admitted for superficial partial thickness burns that occurred less than 72 hours ago [9]. Both groups’ wounds were cleaned with water and 2% chlorhexidine gluconate and provided with ketamine with or without midazolam for anesthesia during the first dressing. In the test group, which included 15 patients, an AFS graft was applied and bandaged. If the AFS graft did not appropriately adhere to the wound, then it was changed. The AFS graft was only removed when there were signs of complete re-epithelization [9]. The control group, which included 15 patients, was treated with a layer of silver sulfadiazine cream 1% and had daily dressing changes. The difference in average number of days for complete re-epithelialization in both groups was not significant, 10.07 ± 0.46 in the test group and 10.47 ± 0.74 in the control group. On the day the dressing was removed, the level of wound improvement was evaluated in both groups with both groups showing that there was a significant improvement in comparison to when the treatment was first started [9]. When it came to pain, there was no difference in how much PO dipyrone, IV tramadol, IV fentanyl, or IV midazolam was needed for pain management in either group. However, there was a difference in the amount of IV ketamine needed with silver sulfadiazine (150.07 ± 70.14) and AFS grafts (76.73 ± 39.12). When it comes to the number of dressing changes, there was a significant difference between the test and control groups [9]. The test group required only 3.00 ± 0.76 dressing changes in comparison to the control group, which required 0.27 ± 1.39 dressing changes [9]. When it comes to the healing time, the results of this study do not support previous studies; however, it does conclude that the use of AFS grafts aids in decreasing the amount of analgesics required and limits the amount of dressing changes which benefits both the patient and their healthcare provider [9].

A randomized controlled trial was conducted by Lima Junior et al. in 2020 to compare the outcomes of superficial and deep partial thickness burns when treated with AFS grafts or silver sulfadiazine cream 1% [10]. A total of 62 patients were divided into three groups. Group One had ten patients in the control group and 13 patients in the test group that were treated in the outpatient setting and had superficial partial thickness burns. Group Two had ten patients in the control group and nine patients in the test group that were treated in the inpatient setting and had more extensive superficial partial thickness burns (between 10%-20% of total body surface area). Group Three had ten patients in the control group and ten patients in the test group that were treated in the inpatient setting and had deep partial thickness burns (5-15% of total body surface area) [10]. The patients were then randomly categorized into a silver sulfadiazine cream 1% group or an AFS graft group. The patients’ wounds were cleaned with water and 2% chlorhexidine gluconate and either had an application of silver sulfadiazine cream 1% or an AFS graft [10]. Outpatient treatment with silver sulfadiazine cream involved dressing changes every two days while inpatient treatment involved daily dressing changes [10]. The number of days required to complete re-epithelialization are as follows: control group one averaged 11.20 ± 0.63 days, test group one averaged 9.77 ± 0.83 days, control group two averaged 11.70 ± 0.67 days, test group two averaged 10.56 ± 1.13 days, control group three averaged 21.30 ± 1.42 days, and test group three averaged 18.10 ± 0.99 days [10]. Pain level was reported to be lower in test groups two and three but there was no significant difference in test group one as compared to the control groups. Pain management requirements presented similar results as well [10]. There was a significant reduction in the number of dressing changes required in all the test groups in comparison to the control groups. Control group one required 5.80 ± 0.42 dressing changes while test group one required 2.08 ± 0.28 dressing changes. Control group two required 11.00 ± 0.47 dressing changes while test group two required 2.33 ± 0.71 dressing changes. Control group three required 20.20 ± 1.69 dressing changes while test group three required only 6.10 ± 2.02 dressing changes [10]. Lima Junior et al. concluded that the use of AFS grafts in wound management proved to show faster wound healing, decreased analgesia requirements, and less frequent dressing changes [10].

In 2021, Lima Junior et al. conducted a larger randomized controlled trial to compare the use of silver sulfadiazine cream 1% to AFS grafts for the treatment of superficial partial thickness burns in the outpatient setting [11]. The study included 115 patients with new and untreated superficial partial thickness burns. Fifty-eight patients were randomly chosen to be treated with silver sulfadiazine cream 1% and 57 patients were randomly chosen to be treated with an AFS graft. The number of days to achieve re-epithelialization, the amount of dressing changes required, and the cost of treatment were the focus of the study. Patients treated with the AFS graft showed re-epithelialization in 9.7 ± 0.6 days in comparison to 10.2 ± 0.9 days in the control group [11]. The AFS group required only 1.6 ± 0.7 dressing changes in comparison to 4.9 ± 0.5 in the control group. Finally, the cost of treatment with AFS grafts was decreased by 42.1% for each patient [11]. These results support the idea that AFS grafts are an excellent choice for burn wound management.

Studies Comparing AFS Grafts With Collagen Alginate Dressings

Winters et al. conducted a retrospective comparative cohort study to evaluate the cost-effectiveness of treating DFUSs with AFS grafts in comparison to collagen alginate dressings in 2020 [12]. A total of 59 DFUs in 55 patients were included in the study and two identical hypothetical cohorts were created, one that treated these wounds with AFS grafts and another that treated them with collagen alginate dressing and the models were then compared [12]. The model showed that the cost of each DFU treatment with AFS grafts was $11,210 in comparison to $15,075 for wounds treated with collagen alginate dressing [12]. There was a higher number of healed wounds and a lower number of amputations associated with the use of AFS grafts. In conclusion, the use of AFS grafts demonstrate a more cost-effective method in the treatment of DFUs while providing better healing outcomes for patients [12].

Lullove et al. performed a randomized controlled trial to compare the efficacy of AFS grafts with collagen alginate dressings in the management of treatment-resistant DFUs in 2021 [13]. A total of 49 patients were included and 25 were randomized to be treated with collagen alginate dressing alone while 24 were treated with the collagen alginate dressing in combination with an AFS graft [13]. All patients underwent two weeks of treatment with offloading using a walker and debridement prior to the start of the trial. The patients were then treated for 12 weeks, and the percentage of complete wound healing was recorded. After six weeks of treatment, the percentage of wound closure was 72.8% in the group treated with AFS grafts but only 41.2% in the group treated with collagen alginate dressing alone. After 12 weeks, results show that 16 of the 24 patients (or 67%) treated with AFS grafts presented with complete wound healing in comparison to only eight of the 25 patients (or 32%) treated with collagen alginate dressing alone [13]. Lullove et al. conclude that DFUs treated with AFS grafts showed an increased number of fully healed wounds as well as faster healing [13].

A larger randomized controlled trial to assess the effectiveness of AFS grafts in comparison to collagen alginate dressings in the management of treatment-resistant DFUs was performed by Lullove et al. in 2022 [14]. A total of 94 patients were included in the study and randomized into groups with either collagen alginate dressing alone or in combination with AFS grafts. Patients were first treated for two weeks with offloading and debridement [14]. When the patients started their treatments, the group being treated with collagen alginate dressing alone received weekly dressing changes at the clinic and three additional changes at home while patients being treated with AFS grafts received a dressing change only once every week [14]. Patients were followed for 12 weeks and the number of wound closures was recorded. Twenty-nine of forty-six patients (or 63%) treated with AFS grafts showed complete wound healing by 12 weeks while only 15 of 48 (or 31.3%) patients treated with collagen alginate dressing alone showed complete wound healing. These results conclude that the use of AFS grafts in the treatment of resistant DFUs is promising [14]. 

Discussion

AFS grafts have proven to be efficacious in the treatment of several different types of wounds, particularly DFUs and burns, which were included in this review. Its unique properties allow it to adhere well to wound beds, limit the number of dressings required, support pro-inflammatory healing, and promote faster and more complete wound healing, all while being more cost effective than dHACM allografts, collagen alginate dressings, and silver sulfadiazine cream 1% [6,9-11,12].

Michael et al. focused on following 58 DFUs in order to assess the time to complete wound closure [5]. Although there was no control to compare the results with, the study focused on the importance of rapid wound healing in the case of DFUs in order to find a treatment that minimizes the amount of amputations seen in patients with treatment-resistant DFUs [5]. The results, however, are in line with the three articles in this review that compared AFS grafts with collagen alginate dressings in the treatment of DFUs. Micheal et al., Winters et al., and the studies done in 2021 and 2022 by Lullove et al. found that the use of AFS grafts for the treatment of DFUs resulted in faster healing and an increased number of wound closures in comparison to the collagen alginate dressings [5,12-14]. There were also no incidents of hypersensitivity reactions to the AFS grafts in any of these studies [5,12-14]. Winters et al. particularly focused on the treatment-related costs of AFS grafts and attributed the lower costs in comparison to collagen alginate dressings to be due to the quicker healing and more complete wound closures [12]. Decreased cost of treatment with AFS grafts was also seen among the studies conducted by Winters et al. and the two studies done in 2021 and 2022 by Lullove et al [12-14].

The studies done by Kirsner et al., Stone II et al., and Wallner et al. focused on comparing the use of AFS grafts with other CTPs, including one synthetic CTP, and all these studies found that the use of AFS grafts in the treatment of wounds showed quicker re-epithelization and ultimately faster wound healing [6-8]. Krisner et al. found that the number of patients who experienced adverse effects such as erythema, pain, and pruritus was not significantly different between the patients treated with AFS grafts and those treated with dHACM [6]. However, this contrasts with what was found in the study utilizing STSG and Suprathel where it was seen that patients treated with AFS grafts reported less pain and pruritus [8]. A unique feature of the study conducted by Wallner et al. was that they tested the level of sebum and skin elasticity in healed wounds 12 months after treatment and compared it to what would normally be found in healthy, non-injured skin [8]. Their findings suggest that wounds treated with AFS grafts revealed better long-term functionality and were aesthetically superior in comparison to the wounds treated with STSG or Suprathel [8].

Finally, the studies conducted by Lima Junior et al. in 2020, 2021, and 2022 focused on the efficacy of AFS grafts in comparison to treatment with silver sulfadiazine cream 1% on burn wounds [9-11]. In all three studies, it was concluded that AFS grafts integrated into the wound better and supported faster healing while requiring less analgesia requirements and fewer dressing changes. The study conducted in 2021 also found that the use of AFS grafts was more cost effective than using silver sulfadiazine in burn wound management [11].

There were several limitations found in the literature reviewed. All studies included in this review had small sample sizes and further testing with a larger cohort may aid in understanding the effects of AFS grafts on wound healing across a variety of wounds. The study conducted by Micheal et al. had a wide inclusion criteria in order to mimic what is likely to be seen in clinical settings and it was recommended that patients follow up weekly or biweekly to assess the wound and measure the healing progress [5]. However, if patients missed appointments but followed up on the 16th week, they were still included in the study. Finally, the studies conducted by Kirsner et al. and Stone II et al. assessed wounds for a mere 62 and 28 days respectively [6,7]. These wounds could have been followed for a longer period of time to properly evaluate scarring and functionality months later. Overall, these studies show great promise in the use of AFS grafts for wound treatment; however, further testing is recommended to confirm these findings in other types of acute and chronic wounds.

## Conclusions

This review of literature demonstrated that the use of AFS grafts on DFUs and burn wounds is superior to many other wound dressing methods. It is understood that the unique biochemical properties of AFS is what makes it an excellent choice for treating chronic and acute superficial and deep partial thickness wounds. The existing research available today is helpful for wound care specialists as it offers a newer and cost-effective alternative with proven excellent outcomes that may benefit both first-world countries and low-income nations. Future randomized controlled trials with large cohorts is recommended to further demonstrate the unique qualities of AFS grafts on wound healing. 
